# Nonprostate Cancer Deaths Best Predict Long-Term Survival in Men Aged ≤59 Years Treated With Low Dose Rate Brachytherapy With or Without Supplemental Therapies: A Mandate for Health Enhancement

**DOI:** 10.1016/j.adro.2026.102101

**Published:** 2026-06-01

**Authors:** Peter Orio, Grgur Mirić, Robert Galbreath, Shalini Moningi, Ryan Fiano, Kent Wallner, Martin King

**Affiliations:** a Dana-Farber Cancer Institute, Boston, Massachusetts; b Urologic Research Institute, Sarasota, Florida; c Bethany College, Bethany, West Virginia; d Ohio University Eastern, St Clairsville, Ohio; e University of Washington, Seattle, Washington

## Abstract

**Purpose:**

Despite favorable biochemical and functional outcomes in younger patients treated with prostate brachytherapy, overall survival remains compromised by a plethora of nonprostate cancer deaths, especially those attributable to sedentary lifestyle or modifiable health risks. In this study, we evaluate cancer control and patterns of death and propose recommendations for lifestyle changes and aggressive management of medical comorbidities.

**Methods and Materials:**

A total of 782 consecutive men aged ≤59 years underwent brachytherapy with or without supplemental therapies. Patients were stratified into 2 age cohorts: ≤54 years (n = 331) and 55 to 59 years (n = 451). Postimplant dosimetry was based on the day 0 computed tomography evaluation. Biochemical failure (BF) was defined as prostate-specific-antigen (PSA) > 0.40 ng/mL after nadir. Patients with metastatic prostate cancer or nonmetastatic castrate-resistant disease who died of any cause were classified as deaths from prostate cancer. All other deaths were attributed to the immediate cause of death. Multiple clinical, pathologic, and treatment parameters were evaluated for their impact on survival.

**Results:**

No significant differences in presentation were observed between the 2 cohorts, except that hypertension and tobacco use were statistically more common in the 55- to 59-year-old age group. The median follow-up was 12.1 years. A total of 40.8% of patients presented with unfavorable intermediate- or high-risk disease. The median day 0 minimum dose of radiation to 90% of the prostate gland (D90) was 122.1%. For the entire cohort, the 15-year BF, prostate cancer-specific mortality, and overall mortality (OM) rates were 4.9%, 1.8%, and 16.5%, respectively. When stratified by age, the 15-year BF rates were 6.1% and 4.0% in the younger and older cohorts, respectively (*p* = .205). The 15-year OM rates were 11.6% and 20.1% in the ≤54- and 55- to 59-year-old groups, respectively (*p* = .002). Prostate cancer accounted for 12.3% of all deaths, with cardiovascular disease and other malignancies comprising 61.4%. In multivariate analysis (MVA), BF was best predicted by high risk (subdistribution hazard ratio [sHR], 10.180; *p* ≤ .001) and percent positive biopsies (sHR, 1.024; *p* = .004), prostate cancer-specific mortality by percent positive biopsies (sHR, 1.030; *p* = .024), and OM by age (hazard ratio [HR], 1.105; *p* = .002), diabetes (HR, 1.890; *p* = .036), and current tobacco use (HR, 2.989; *p* < .001).

**Conclusions:**

Brachytherapy generates favorable oncologic outcomes in younger men with prostate cancer. Nonprostate cancer deaths substantially outnumber prostate cancer deaths, with the majority due to cardiovascular disease or other malignancies. Improvement in overall survival will require multiple lifestyle changes and aggressive management of modifiable health risks.

## Introduction

In the United States, the widespread implementation of prostate-specific-antigen (PSA) screening and increased public awareness of prostate cancer have resulted in stage migration and a decrease in patient age at diagnosis, with 10% of all prostate cancer diagnoses in men aged ≤55 years.^1^ Younger patients with long life expectancies are most likely to benefit from therapeutic interventions that maximize long-term cancer control with favorable quality-of-life (QOL) outcomes. Multiple brachytherapy studies have documented both favorable biochemical and functional outcomes in younger patients.^2^, ^3^, ^4^, ^5^, ^6^ Despite these reported high cure rates, overall survival remains compromised by a plethora of nonprostate cancer deaths, especially those attributable to modifiable health risks such as cardiovascular disease (CVD), obesity, tobacco use, and sedentary lifestyles. Healthy lifestyles are associated with a substantial decrease in prostate cancer and nonprostate cancer deaths.^7^ In this study, we evaluate the durability of our previously reported biochemical control rates^2^ in younger patients (stratified into cohorts ≤54 years and 55-59 years), as well as prostate cancer-specific mortality (PCSM), overall mortality (OM), and patterns of death, and provide recommendations for lifestyle changes and aggressive medical management of modifiable health risks.

## Methods and Materials

From April 1995 through November 2018, 782 consecutive patients aged ≤59 years with National Comprehensive Cancer Network (NCCN) low-, intermediate-, and high-risk disease underwent low-dose rate prostate brachytherapy (92.7% were implanted with Pd-103) with or without supplemental therapies. Patients were stratified into 2 age cohorts (≤54 years and 55-59 years) to detect any age-related differences in presentation or outcomes. The analysis was conducted with institutional review board approval. Patients were clinically staged using medical history and physical examination, including a digital rectal examination and PSA. Beginning in October 2001, serum testosterone was added to the work-up. Bone and computed tomography (CT) scans of the chest/abdomen/pelvis were obtained for all higher-risk patients at the discretion of either the referring or treating physician. All biopsy slides were reviewed by a single pathologist with expertise in prostate cancer.

The brachytherapy target volume consisted of the prostate gland with 5 mm periprostatic margins (except posteriorly, where there was no margin) and the proximal 10 to 12 mm margins of the seminal vesicles.^8^ This planning philosophy resulted in a target volume approximately 1.9 times the actual prostate size. All postimplant dosimetric calculations were based on the day 0 CT evaluation. Prior to 2000, a preplanned approach was utilized. Subsequently, preplanning served as a template for intraoperative dosimetry, with the goal of achieving a postimplant target volume receiving a minimum dose of radiation to 90% of the prostate gland (D90) of approximately 120% and a mean urethral dose of approximately 110% of the prescription dose. Implant prescription doses were 125 Gy and 145 Gy for Pd-103 and I-125 monotherapy, respectively, and 90 Gy and 110 Gy for the Pd-103 and I-125 boost, respectively. All brachytherapy was performed by a single brachytherapist (G.M.).

For patients receiving supplemental external beam radiation therapy (EBRT), the target volume consisted of the prostate gland and seminal vesicles with a margin. In all high-risk and selected unfavorable intermediate-risk (UIR; depending on PSA, Gleason score, clinical stage, and percent positive biopsies) patients, the pelvic lymph nodes were also included. Supplemental EBRT was administered using either a 3-dimensional conformal technique or intensity modulated EBRT prior to the brachytherapy boost. The brachytherapy boost was routinely delivered 4 to 15 days following completion of EBRT. When prescribed, androgen deprivation therapy (ADT) was initiated 3 months prior to implantation and consisted of a luteinizing hormone/releasing hormone agonist and an antiandrogen or a luteinizing hormone/releasing hormone antagonist. ADT was administered either for prostate downsizing before implantation or as treatment intensification for patients with higher-risk disease characterized by adverse pathologic features.

Patients were monitored by physical examination, including digital rectal examination, and by PSA and testosterone measurements (after October 2001) at 3- to 6-month intervals. The endpoints of the analysis were biochemical failure (BF), PCSM, OM, and patterns of death. BF was defined as a PSA > 0.40 ng/mL after nadir. Patients who failed to achieve a PSA ≤ 0.40 ng/mL were categorized as BFs.^2^^,^^3^ The cause of death was determined for each deceased patient. Patients with metastatic prostate cancer or nonmetastatic castrate-resistant disease who died of any cause were classified as deaths from prostate cancer. All other deaths were attributed to the immediate cause of death. All patients were followed until death. The cause of death was determined by a review of departmental, hospital, and primary care records along with death certificates.

Patients were grouped according to age (≤54 years and 55-59 years). The clinical, treatment, and dosimetry values collected as continuous variables were compared using an independent *t* test. Variables collected as categorical were presented using contingency tables. Pearson’s Chi-suare test and Fisher’s exact test were used to determine if the distributions of these categorical variables were different between the 2 age groups. OM was compared between the 2 age groups and across NCCN risk groups (4 groups) using Cox regression analysis. Univariate analyses were used to estimate hazard ratios (HRs) of selected variables for OM. Variables with *p* values < .10 were entered into the multivariate analysis. BF and PCSM rates across the different age groups and risk levels were estimated using a competing risks (Fine-Gray) analysis and presented as cumulative incidence rates. Univariate analyses were used to estimate the subdistribution hazard ratios (sHRs) of selected variables for BF and PCSM. Variables with *p* values < .10 were entered into the multivariate analysis. STATA version 18.0 (StataCorp LLC) and SPSS version 29.0 (IBM) were used for analyses, with significance set at *p* < .05.

## Results

Table 1 summarizes the clinical, treatment, and dosimetric parameters of the 782 patients (age range, 35-59 years), stratified by age ≤54 years (n = 331) and 55 to 59 years (n = 451). No significant differences in presentation between the 2 cohorts were observed, except that hypertension (*p* = .02) and tobacco consumption (*p* = .043) were statistically more common among older patients. The median follow-up was 12.1 years. A total of 40.8% of patients presented with unfavorable intermediate risk (UIR) or high-risk disease. The median day 0 postimplant D90 was 122.1%. Seven hundred twenty-five patients (92.7%) were implanted with Pd-103. EBRT was administered to 50.6% of patients with pelvic lymph nodes within the target volume among high-risk and selected UIR patients. ADT was administered to 19.1% of patients, with a median duration of 4 months in the ≤6-month group and 12 months in the >6-month group (maximum duration, 36 months). For all biochemically controlled patients, the most recent median PSA was <0.01 ng/mL.

**Table 1:** Clinical, dosimetric, and treatment parameters, stratified by age (≤54 years and 55-59 years)

	≤54 y (n = 331)			55-59 y (n = 451)			Total (N = 782)			
Variable	Median	Mean	SD	Median	Mean	SD	*P* value	Median	Mean	SD
Age (y)	51.0	50.0	3.1	57.0	57.2	1.4	<.001	55.0	54.5	3.9
Follow-up	12.2	12.0	5.3	11.7	11.6	5.5	.273	12.1	11.8	5.5
Pretreatment PSA	5.7	8.0	9.8	5.6	7.9	8.4	.946	5.6	7.9	9.0
Positive biopsies (%)	33.3	36.8	26.4	30.0	37.2	25.2	.835	33.3	37.0	25.7
BMI (kg/m^2^)	28.7	29.4	5.0	28.4	29.6	5.3	.667	28.5	29.5	5.2
Prostate volume	27.3	27.9	7.8	29.1	29.2	8.7	.058	28.4	28.6	8.4
Average urethral dose (%)	113.9	114.9	12.3	13.2	113.6	12.3	.139	113.4	113.2	12.3
V100	98.4	97.7	2.7	98.3	97.5	2.9	.397	98.3	97.6	2.8
V150	73.5	72.2	10.0	72.8	70.9	10.8	.089	73.1	71.4	10.5
V200	43.8	42.8	9.6	42.9	41.8	10.2	.142	43.1	42.2	10.0
D90 (%)	122.4	122.8	11.1	121.7	121.6	10.9	.143	122.1	122.1	11.0
Most recent PSA	<0.01	0.02	0.07	<0.01	0.02	0.08	.940	<0.01	0.02	0.08

	n (%)			n (%)			*P* value	n (%)		
PSA (ng/mL)							.892			
≤10	274 (82.8)			375 (83.2)				649 (83.0)		
>10	57 (17.2)			76 (16.9)				133 (17.0)		
Gleason group							.051			
1	142 (42.9)			186 (41.2)				328 (41.9)		
2	111 (33.5)			133 (29.5)				244 (31.2)		
3	48 (14.5)			74 (16.4)				122 (15.6)		
4	11 (3.3)			37 (8.2)				48 (6.1)		
5	19 (5.7)			21 (4.7)				40 (5.1)		
Clinical stage							.361*			
T1b-T2a	311 (94.0)			412 (91.4)				723 (92.5)		
T2b-T2c	19 (5.7)			35 (7.8)				54 (6.9)		
T3	1 (0.3)			4 (0.9)				5 (0.6)		
NCCN risk category							.189			
Low	133 (40.2)			165 (36.6)				298 (38.1)		
Favorable intermediate	76 (23.0)			89 (19.7)				165 (21.1)		
Unfavorable intermediate	86 (26.0)			128 (28.4)				214 (27.4)		
High	36 (10.9)			69 (15.3)				105 (13.4)		
Isotope							.972			
Pd-103	307 (92.8)			418 (92.7)				725 (92.7)		
I-125	24 (7.3)			33 (7.3)				57 (7.3)		
ADT duration (mo)							.187			
0	274 (82.8)			359 (79.6)				633 (81.0)		
≤6	15 (4.5)			35 (7.8)				50 (6.4)		
>6	42 (12.7)			57 (12.6)				99 (12.7)		
EBRT	165 (49.9)			231 (51.2)			.705	396 (50.6)		
Hypertension	120 (36.3)			201 (44.6)			.020	321 (41.1)		
Diabetes	21 (6.3)			36 (8.0)			.384	57 (7.3)		
Coronary artery disease	23 (7.0)			45 (10.0)			.137	68 (8.7)		
Hypercholesterolemia	92 (27.8)			123 (27.3			.872	215 (27.5)		
Tobacco							.043			
Never	156 (47.1)			181 (40.1)				331 (43.0)		
Former	91 (27.5)			161 (35.2)				252 (32.2)		
Current	84 (25.4)			109 (24.3)				193 (24.7)		
Testosterone (n = 488)							.486			
Low & lower: 1/3 normal	187 (71.4)			257 (73.6)				444 (72.7)		
Middle: 1/3 normal	50 (19.1)			68 (19.5)				118 (19.3)		
Upper: 1/3 normal & high	25 (9.5)			24 (6.9)				49 (8.0)		
PNI	100 (30.2)			131 (29.1)			.724	231 (29.5)		

*Abbreviations:* ADT = androgen deprivation therapy; BMI = body mass index; EBRT = external beam radiation therapy.

⁎ Fisher’s exact test.

**Figure 1:**
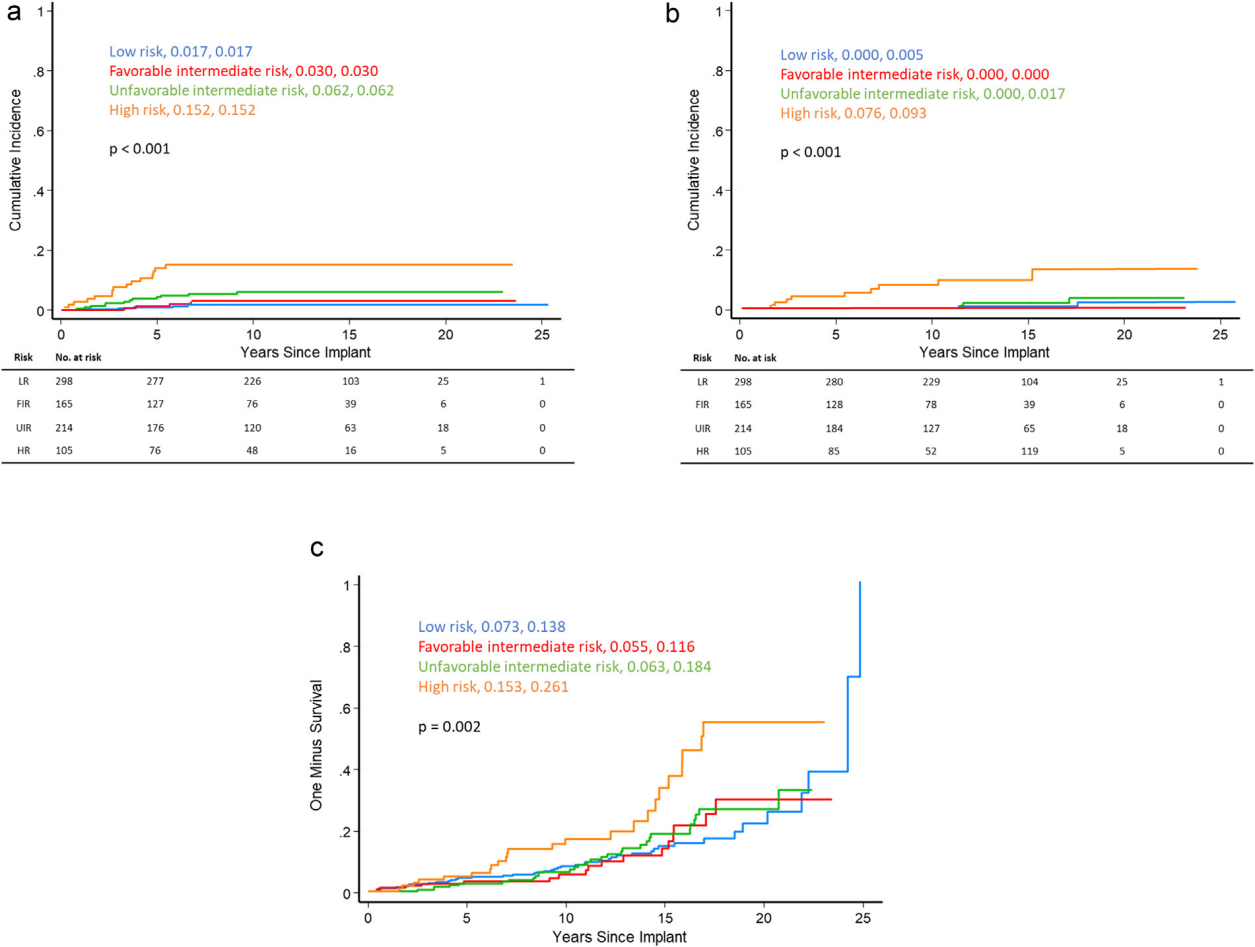
(a) Biochemical failure at 10 and 15 years, stratified by risk. (b) Prostate cancer-specific mortality at 10 and 15 years, stratified by risk. (c) Overall mortality at 10 and 15 years, stratified by risk.

**Figure 2:**
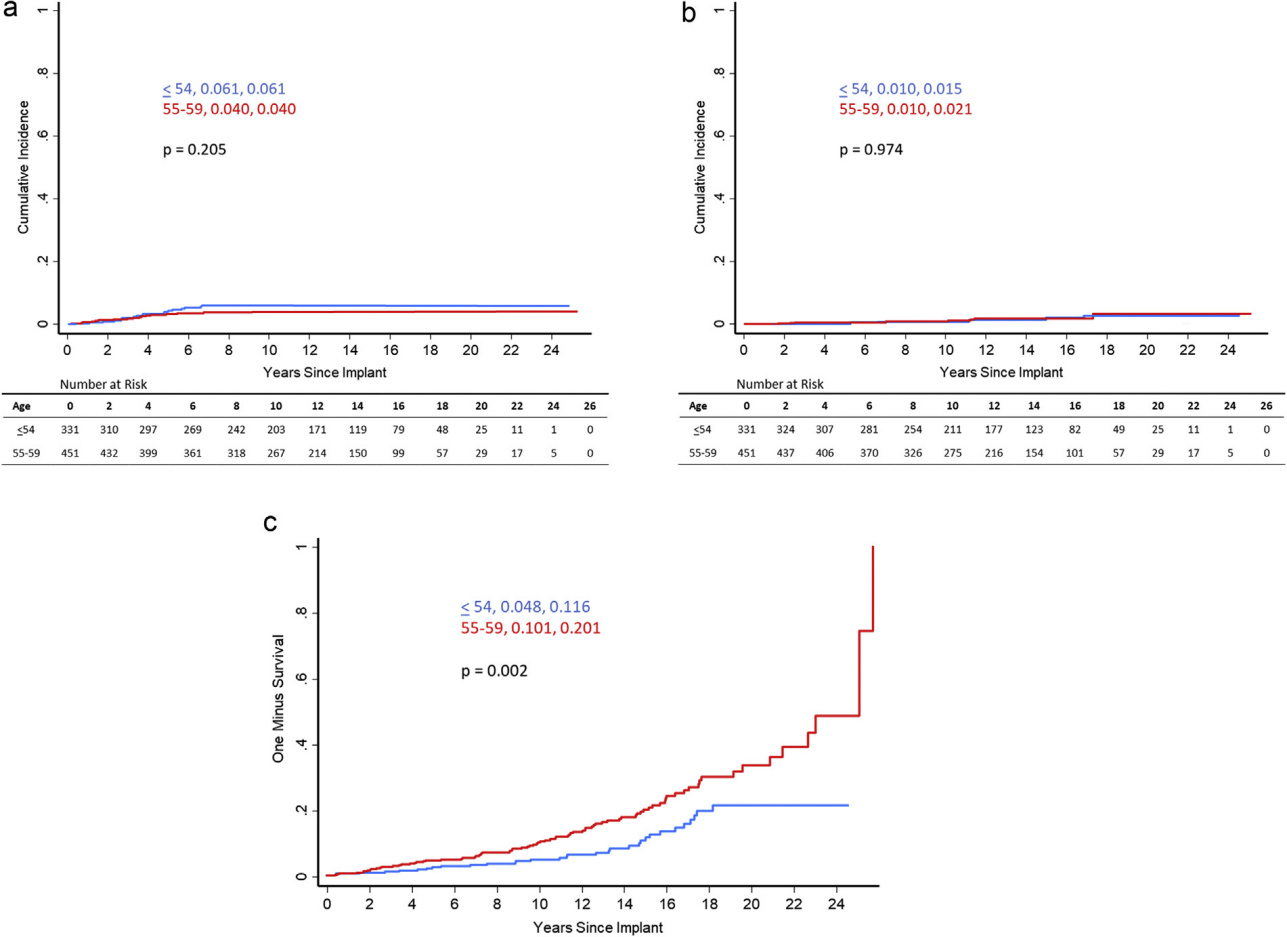
(a) Biochemical failure at 10 and 15 years, stratified by age group. (b) Prostate cancer-specific mortality at 10 and 15 years, stratified by age group. (c) Overall mortality at 10 and 15 years, stratified by age group.

For the entire cohort, the 15-year BF, PCSM, and OM rates were 4.9%, 1.8%, and 16.5%, respectively. Evaluation by risk group revealed significant differences in BF (*p* < .001), PCSM (*p* < .001), and OM (*p* = .002) rates, with only a small fraction of deaths due to prostate cancer (Fig. 1a-c). When stratified by patient age, BF rates were 6.1% and 4.0% in the younger and older patients, respectively (*p* = .205; Fig. 2a). While no statistical difference in PCSM (1.5% vs 2.1%; *p* = .974; Fig. 2b) was noted, a statistically significant difference in OM between the 2 groups was identified (*p* = .002; Fig. 2c). In both age cohorts, OM approximately doubled from years 10 to 15 (4.8% to 11.6% in younger patients and 10.1% to 20.1% in older patients [55-59-year-old group]). Consistent with the age differential (median age, 51.0 vs 57.2 years) at presentation, the 15-year OM rate in the ≤54-year-old group approximated the 10-year OM rate in the 55- to 59-year-old group.

**Figure 3:**
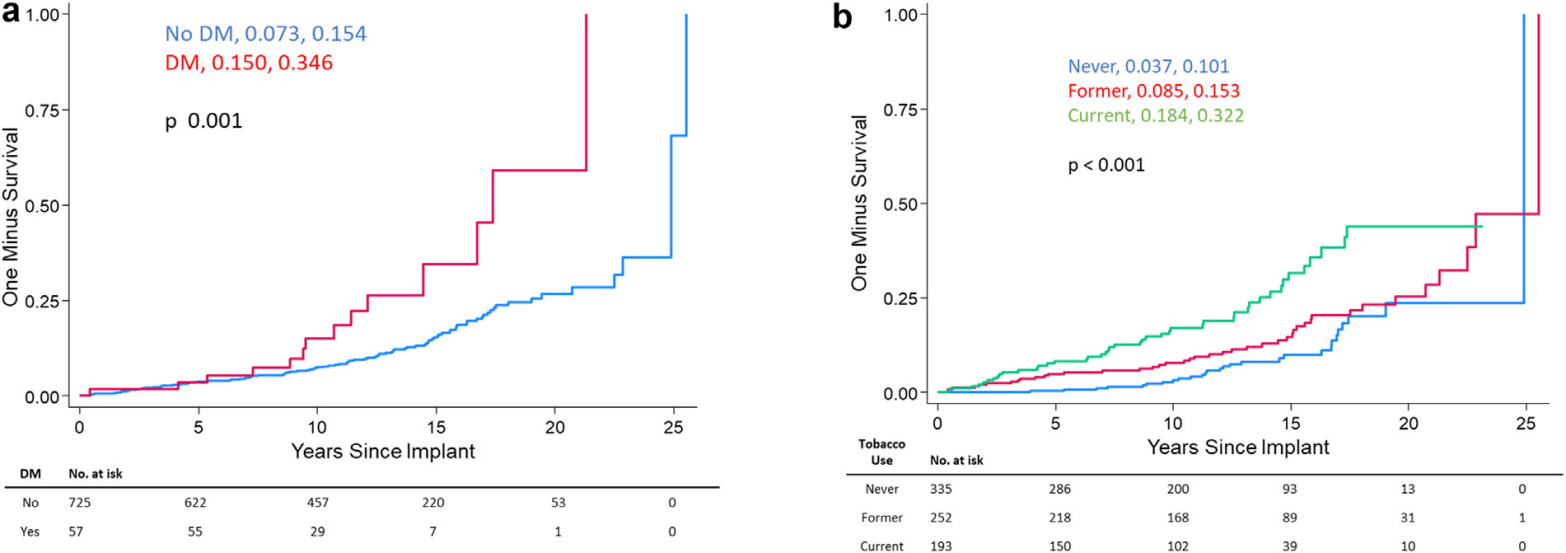
(a) Overall mortality at 10 and 15 years, stratified by diabetes mellitus (DM). (b) Overall mortality at 10 and 15 years, stratified by tobacco use.

Patterns of death demonstrated a preponderance of nonprostate cancer deaths in each group (Table 2). Overall, 114 patients died. Of these, prostate cancer was responsible for 14 deaths (12.3%), while CVD (31.6%) and other malignancies (29.8%) accounted for 61.4% of all deaths. The first 10 years following implantation accounted for half of the prostate cancer deaths and all 3 of the bladder, rectum, and colon cancer deaths (1 death per site). All 3 patients were treated with monotherapeutic brachytherapy and died 3.5 years (rectum), 3.1 years (bladder), and 8.1 years (colon) following therapy. The incidence of death from CVD and other malignancies remained relatively constant throughout the duration of follow-up. Chronic health conditions adversely impacted long-term survival. The impact of modifiable health risks, such as diabetes and tobacco use, on OM is illustrated in Fig. 3a, b. Patients with diabetes were more than twice as likely (34.6% vs 15.4%) to have died at 15 years than those without such a diagnosis. While current tobacco smokers had a 32.2% death rate at 15 years, former smokers approached a death rate of never smokers (15.3% vs 10.1%).

**Table 2:** Causes of death, stratified by years

Patterns of death	<10 y from implant n (%)	10-15 y from implant n (%)	>15 y from implant n (%)	Total n (%)
Prostate cancer	7 (13.2)	4 (12.1)	3 (10.7)	14 (12.3)
Cardiovascular disease	19 (35.9)	11 (33.3)	6 (21.4)	36 (31.6)
Renal failure	0 (9)	3 (9.1)	3 (10.7)	6 (5.3)
Sepsis	0 (0)	3 (9.1)	3 (10.7)	6 (5.3)
Rectal cancer	1 (1.9)	0 (0)	0 (0)	1 (0.9)
Colon cancer	1 (1.9)	0 (0)	0 (0)	1 (0.9)
Bladder cancer	1 (1.9)	0 (0)	0 (0)	1 (0.9))
Lung cancer	7 (13.2)	5 (15.2)	4 (14.3)	16 (14.0)
Aerodigestive (esophagus, tonsil)	3 (5.7)	0 (0)	1 (3.6)	4 (3.5)
Other cancers	5 (9.4)	3 (9.1)	3 (10.7)	11 (9.7)
Other causes	9 (17.0)	4 (12.1)	5 (17.9)	18 (15.8)

In the Fine-Gray analysis, BF was best predicted by high risk (sHR, 10.180; 95% CI, 2.603-39.815; *p* = .001) and percent positive biopsies (sHR, 1.024; 95% CI, 1.008-1.041; *p* = .004), and PCSM by percent positive biopsies (sHR, 1.030; 95% CI, 1.004-1.056; *p* = .024). OM was most closely associated with age (HR, 1.105; 95% CI, 1.038-1.176; *p* = .002), diabetes (HR, 1.890; 95% CI, 1.042-3.425; *p* = .036), and current tobacco use (HR, 2.989; 95% CI, 1.832-4.873; *p* < .001). ADT did not impact OM (HR, 1.214; 95% CI, 0.752-1.958; *p* = .427). In the subgroup analysis, there was no association between ADT, diabetes, and OM (*p* = .477). However, ADT interacted with body mass index (BMI), with the association restricted to men with a baseline BMI ≥ 30 kg/m^2^. In that BMI cohort, ADT use increased the 15-year OM rate from 18.9% to 35.0% (*p* = .027).

## Discussion

With the widespread implementation of PSA screening and resultant greater public awareness, the diagnosis of prostate cancer has become increasingly common in younger patients, with 10% of all new diagnoses in men aged ≤55 years.^1^ Younger patients’ long life expectancy makes them most likely to benefit from effective therapeutic interventions that maximize cure and preserve QOL. Multiple monotherapy and boost brachytherapy studies have reported outstanding biochemical cure rates, favorable functional outcomes, and low PCSM across all risk groups, Gleason scores, and patient ages.^2^, ^3^, ^4^, ^5^, ^6^^,^^9^^,^^10^ Our current study confirms the durability of biochemical control and low PCSM in a large series of younger men with mature follow-up (median, 12.1 years). Although we are encouraged by the fact that the vast majority of BFs occurred within the first 7 years following treatment and that PCSM increased from 0% to 1.7% between years 10 and 15 (depending on the risk group) (Fig. 1a, b), the young age of these patients necessitates additional follow-up to confirm the durability of oncologic outcomes. Radiation-induced malignancies are a concern in patients with a prolonged life expectancy. Radiation therapy accounts for approximately 5% of all second malignancies, with an overall incidence of 0.03% to 0.3%.^11^ Our database documents deaths from second malignancies, but not the incidence of such events. Three monotherapeutic brachytherapy patients died of possible radiation-induced malignancies (1 rectum, 1 bladder, and 1 colon), but the deaths occurred 3.5, 3.1, and 8.1 years following treatment, which is too short an elapsed period of time to establish a causal relationship. The efficacy of brachytherapy depends on high-quality dose distributions, dose escalation that is not achievable with other radiation modalities, and the ability to generously treat the periprostatic margins and proximal seminal vesicles.^3^^,^^12^ However, because of the long natural history of prostate cancer, biochemical control alone is insufficient to maximize optimal patient outcomes. In prostate cancer, overall survival is compromised by a plethora of nonprostate deaths, with a preponderance due to CVD and other malignancies. Healthy lifestyles are associated with a decreased risk of prostate cancer and nonprostate cancer deaths.^7^ Plym et al^13^ reported that 36% of prostate cancer deaths in men with a high genetic risk of prostate cancer are preventable with a heart-healthy lifestyle.

The identification of patients at significant cardiovascular risk is essential because CVD is highly treatable with improvement in overall survival. Consistent with prior prostate cancer series, CVD was the number one cause of death in the present study. A total of 31.6% of all deaths were due to CVD, with the incidence being relatively constant through 15 years. The impact of modifiable health risks on OM, including CVD, is illustrated in Fig. 3. Unfortunately, clinicians have not adequately assessed patients for cardiovascular risk. In a large United States Veterans study of men with prostate cancer, 2 out of 3 were at high cardiovascular risk, more than 50% presented with uncontrollable cardiovascular risk factors (blood pressure, cholesterol, and glucose), and 1 out of 3 did not have a comprehensive cardiovascular risk assessment.^14^ In contrast, the PRONOUNCE trial (which randomized patients with known CVD to 2 different ADT regimens) mandated universal cardiology management.^15^ The trial reported significantly lower-than-expected rates of major adverse cardiac events in both arms and provided evidence to support the routine inclusion of cardiology in the management of patients with prostate cancer. In order to mitigate the impact of CVD, Atkins and Nikolova^16^ propose a baseline assessment followed by annual cardiovascular risk assessments. Cardiology management is recommended for men aged >60 years with at least 1 cardiovascular risk factor, men aged <60 years with 2 or more cardiovascular risk factors, or a strong family history of atherosclerotic CVD. Optimal management includes aggressive medical management of hypertension, diabetes, hypercholesterolemia, and obesity (especially central adiposity); a high-fiber/low-fat, heart-healthy diet; tobacco cessation; and routine physical activity, including aerobic exercise and resistance training. Finally, adherence to colorectal screening protocols is essential and likely explains the small number of colorectal cancer deaths in our series (Table 2).

Routine aerobic exercise and resistance training are essential for improving cardiovascular fitness, maintaining optimal body weight, maximizing muscle mass, and minimizing obesity. Moore et al^17^ reported that 150 minutes of exercise/wk improved cardiovascular status, reduced all-cause mortality, and reduced the incidence of 13 cancers, although prostate cancer was not among them. Exercise has been shown to reduce prostate cancer deaths by 30% to 50% and OM by up to 50%.^7^^,^^13^^,^^18^ In particular, adherence to postcancer diagnosis exercise regimens reduces PCSM and all-cause mortality.^18^^,^^19^ Resistance training is also essential for preserving bone integrity, especially in ADT-treated patients. In addition, in our present study, ADT negatively impacted survival in men with a BMI ≥ 30 kg/m^2^. We recommend consultation with an exercise physiologist and a dietitian to formulate and implement strategies specific to the needs of the individual patient.

Consistent with the medical literature, this study demonstrates that cigarette smoking adversely impacts overall survival (Fig. 3b). The most effective smoking cessation protocols combine pharmacologic and behavioral therapy. However, a brief counseling intervention by itself can strongly influence a patient’s decision to permanently stop smoking.^20^^,^^21^ Persistent smoking after diagnosis increases the risk of metastases and fatal prostate cancer,^7^ while the discontinuation of smoking increases overall survival and decreases the risk of a second malignancy.^22^ Individuals who refrain from cigarette consumption for more than 10 years have prostate cancer death rates comparable to those of never smokers.^23^ In addition, the use of tobacco is associated with alcohol consumption. Alcohol abuse corresponds to a higher incidence of overall cancer, increased cancer recurrence, and death, and a higher incidence of treatment and surgical complications.^24^ Immediate cessation of all tobacco products and emphasis on minimizing alcohol consumption should be integrated into routine prostate cancer counseling and management.

Despite marked improvement in biochemical control following all curative prostate cancer treatment modalities and a resultant reduction in PCSM, improvement in overall survival has been elusive. Failure to improve overall survival has been, in part, because prostate cancer physicians have not emphasized survivorship issues, including medical management, cardiology assessments, and lifestyle changes. In summary, we strongly recommend that men engage in daily aerobic exercise and resistance training 3 times/wk. Consultation with an exercise physiologist is preferred to ensure an optimal exercise regimen, provide oversight, and minimize exercise-related injuries. Patients should follow a heart-healthy diet, refrain from all tobacco products, consume alcohol in moderation, and diligently follow medical advice regarding the management of comorbidities and preventive screening procedures, such as colonoscopy, Dual-Energy X-Ray Absorptiometry scans, and chest CT (in long-term smokers).

Strengths of this study include mature follow-up in a relatively large cohort of consecutively treated younger patients, intensive and complete follow-up, and the consistent delivery of monotherapy or boost brachytherapy across all risk groups and Gleason scores by a single physician (G.M.). The major shortcomings are the lack of information on the duration, severity, and management of the evaluated comorbidities, and the absence of QOL assessments.

The next frontier for prostate cancer physicians and patients is the development and implementation of clinical programs focused on improving overall survival through active, longitudinal patient engagement. This will require multidisciplinary collaboration, active participation across all aspects of men’s health, and support for ongoing and future clinical trials.

## Conclusions

Brachytherapy is an efficacious treatment for younger men with prostate cancer. However, overall survival is compromised by a plethora of nonprostate cancer deaths, which outnumber prostate cancer deaths by more than 7-fold, with the majority due to CVD and other malignancies. Improvements in overall survival among these younger men mandate multiple lifestyle changes, including cardiovascular risk assessments, daily physical activity consisting of aerobic and resistance training, a heart-healthy diet, cessation of tobacco use, and aggressive management of modifiable health risks.

## Disclosures

Peter Orio: consulting for Teleflex Inc and Theragenics Corp. Grgur Mirić: consultant for Theragenics. Robert Galbreath: none. Shalini Moningi: none. Ryan Fiano: none. Kent Wallner: none. Martin King: grants or contracts: Bayer (research), Teleflex (research), and AstraZeneca (research); payment or honoraria: Bayer and Pfizer; support for attending meetings: Bayer; participation on a Data Safety Monitoring Board or Advisory Board: Teleflex.
